# Structures of β-glycosidase LXYL-P1-2 reveals the product binding state of GH3 family and a specific pocket for Taxol recognition

**DOI:** 10.1038/s42003-019-0744-4

**Published:** 2020-01-10

**Authors:** Lin Yang, Tian-Jiao Chen, Fen Wang, Li Li, Wen-Bo Yu, Yi-Kang Si, Jing-Jing Chen, Wan-Cang Liu, Ping Zhu, Weimin Gong

**Affiliations:** 1 National Research Laboratory for Physical Sciences in Microscales, University of Science and Technology of China, 230026 Hefei, Anhui China; 2 State Key Laboratory of Bioactive Substance and Function of Natural Medicines and Key Laboratory of Biosynthesis of Natural Products of the State Health Commission, Institute of Materia Medica, Chinese Academy of Medical Sciences and Peking Union Medical College, 1 Xian Nong Tan Street, 100050 Beijing, China

**Keywords:** X-ray crystallography, Nanocrystallography

## Abstract

LXYL-P1-2 is one of the few xylosidases that efficiently catalyze the reaction from 7-β-xylosyl-10-deacetyltaxol (XDT) to 10-deacetyltaxol (DT), and is a potential enzyme used in Taxol industrial production. Here we report the crystal structure of LXYL-P1-2 and its XDT binding complex. These structures reveal an enzyme/product complex with the sugar conformation different from the enzyme/substrate complex reported previously in GH3 enzymes, even in the whole glycohydrolases family. In addition, the DT binding pocket is identified as the structural basis for the substrate specificity. Further structure analysis reveals common features in LXYL-P1-2 and Taxol binding protein tubulin, which might provide useful information for designing new Taxol carrier proteins for drug delivery.

## Abstract (web summary)

Yang, Chen et al. report crystallographic structures of GH3 beta-glucosidase LXYL-P1-2 that converts XDT into DT, a precursor for an anticancer drug taxol, and the complex of LXYL-P1-2 with XDT. This study provides insight into the catalytic mechanism for GH3 enzymes.

## Introduction

**Fig. 1:**
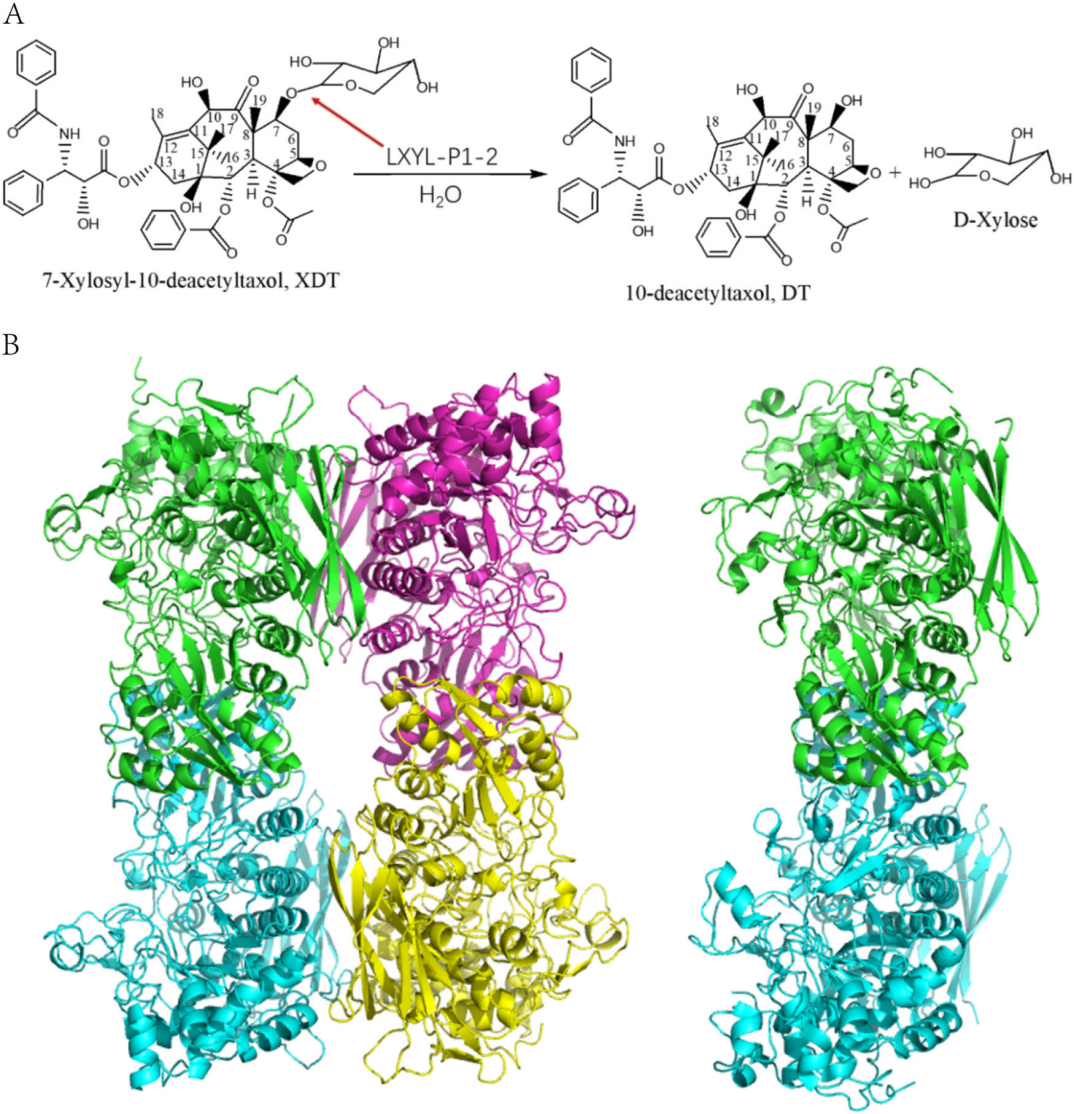
**a** Chemical transformation of XDT to DT with LXYL-P1-2 catalyzed. **b** Tetramer structure of LXYL-P1-2. The four monomers are colored in green, cyan, magenta, and yellow, respectively. The dimer structure of *Aa*BGL1 (PDB code 4IIB). The two monomers are colored in green and cyan and made the same orientation as the dimer in LXYL-P1-2.

Taxol (generic name: paclitaxel), a rare natural product mainly generated by yew bark, is the well-known blockbuster anticancer drug. It promotes tubulin assembly into microtubules and prevents their disassembly^1^. However, the natural level of Taxol is extremely low^2^, while the content of 7-β-xylosyl-10-deacetyltaxol (XDT) can be up to 25 times of Taxol^3^. XDT is often regarded as the waste during Taxol extraction process, causing both resource loss and potential environmental pollution. Compared with Taxol, XDT lacks the C10 hydroxyl group but harbors an additional β-xylosyl group at the C7 position. If the xylosyl group is removed, the resultant 10-deacetyltaxol (DT) can be used as a precursor for Taxol preparation. Therefore, the β-xylosidase catalyzing the removal of xylosyl from XDT (Fig. 1a) would play a prominent role in reducing both of the resource waste and pollution to environment. However, a lot of commercially available β-xylosidases have been demonstrated to possess no activity on releasing the xylosyl residue from XDT^4^. Recently, we identified two bifunctional β-xylosidase/glucosidase (named as LXYL-P1-1 and LXYL-P1-2) from *Lentinula edodes*, which belong to GH3 family and can efficiently convert XDT into DT^5^. By combining LXYL-P1**-**2 and an engineered acetyltransferase, we have constructed an in vitro one-pot reaction system for converting XDT into Taxol^6^.

The catalytic specificity and higher efficiency of LXYL-P1-2 prompted further investigation of its structure-function relationship. Here we present the crystal structures of LXYL-P1-2 and its complex with XDT. The binding mode of xylose group shed lights on the catalytic mechanism for GH3 enzymes. DT binding pocket elucidates the structural basis of substrate specificity. Structural comparison of LXYL-P1-2 and tubulin suggests a possible common feature for designing Taxol binding protein.

## Results

### Structures of LXYL-P1-2 in substrate free form

Highly glycosylated proteins, such as LXYL-P1-2, are greatly difficult to be crystalized. To improve formation of good quality crystals, LXYL-P1-2 was endoglycosidase-treated before crystallization^5^. The crystallographic statistics for data collection and structure refinement are summarized in Table 1. The first 43 residues were missing in the electron density map, partially supporting the existence of the KEX2^7^ cleavage site (Ile^32^Phe^33^Arg^34^Arg^35^). The first amino acid of the mature protein was then verified to be Asp^36^ by N-terminal sequencing.

**Table 1:** Statistics of data-collection and refinement.

Data sets	Native(6JBS)	E529Q-XDT(6KJ0)
Diffraction data		
Space group	*P4*_*3*_*2*_*1*_*2*	*C222*_*1*_
Cell dimensions		
*a, b, c* (Å)	131.9,131.9,385.9	79.9, 182.2,241.5
α, β, γ (°)	90, 90, 90	90, 90, 90
Resolution range (Å)	50-2.4 (2.49–2.40)	50-2.27 (2.30–2.27)
Number of unique reflections	133487	76271
Data completeness (%)	99.9 (99.1)	93.5 (94.1)
Redundancy	12.8 (7.1)	8.6 (8.4)
〈*I*〉/〈σ(*I*)〉	23.6 (2.9)	27.1 (4.0)
*R*_merge_^a^	0.155 (0.667)	0.122 (0.548)
Refinement		
*R-*factor/*R*_free_^b^	0.184/0.251	0.192/0.251
Number of reflections used	126287	72397
r.m.s.d. bond length (Å)	0.009	0.017
r.m.s.d. bond angles (°)	1.527	2.040
Mean *B* factor (Å^2^)		
Protein main-chain atoms	33.9	33.8
Protein side-chain atoms	35.4	34.5
Water molecules	32.9	33.8
Glycan	51.8	55.6
Tris	42.1	–
DT	–	55.1
Xylose	–	34.8
No. of atoms		
Protein	22984	11433
Water molecules	1544	683
Glycan	952	493
Tris	32	–
DT	–	118
Xylose	–	20

The free *R* factor was calculated using 5% of reflections omitted from the refinement

^a^*R*_merge_ = ∑_*hkl*_∑_*i*_ |*I*_*i*_(*hkl*) − 〈*I*(*hkl*)〉|/∑_*hkl*_∑_*i*_ I_*i*_(*hkl*), where 〈*I*(*hkl*)〉 is the main value of *I*(*hkl*)

^b^*R*-factor = ∑||*F*_obs_| − |*F*_calc_||/∑|*F*_obs_|, where Fobs and Fcalc are observed and calculated structure factors

LXYL-P1-2 exists as a 222 symmetric tetramer in the asymmetric unit (Fig. 1b) (PDB code 6JBS), consistent with the molecular weight measured by Gel filtration experiment. The structure of the four monomers are essentially same without any remarkable difference. Each monomer comprises three domains as some GH3 members. Domain 1 folds into a TIM barrel-like structure and contains the residues from Asp^36^ to Gly^365^. Domain 2 (residue 398–600) is an α/β sandwich, in which five parallel β-strands and one antiparallel β-strand are sandwiched by five α-helixes. Domain 1 and domain 2 is connected by linker 1 (residue 366–397). Domain 3 (residue 664–803) is connected to domain 2 by linker 2 (residue 601–663) and has a fibronectin type III (FnIII) fold. Seven Asn residues (81, 272, 342, 385, 457, 576, and 635) are found to link with different types of oligosaccharide even after treated by endoglycosidase H (Table 2).

**Table 2:** Glycan modification of LXYL-P1-2.

Residue	Glycan structure
Asn81	GlcNAc-β1-4-GlcNAc-α1-4-Man
Asn272	GlcNAc-β1-4-GlcNAc-β1-4-Man-α1-4-Man
Asn342	GlcNAc
Asn385	GlcNAc
Asn457	GlcNAc-β1-4-GlcNAc-α1-4-Man-α1-3-Man(-β1-6-Man)-α1-2-Man-α1-2-Man
Asn576	GlcNAc
Asn635	GlcNAc

### Structure of E529Q mutant co-crystallized with XDT

**Fig. 2:**
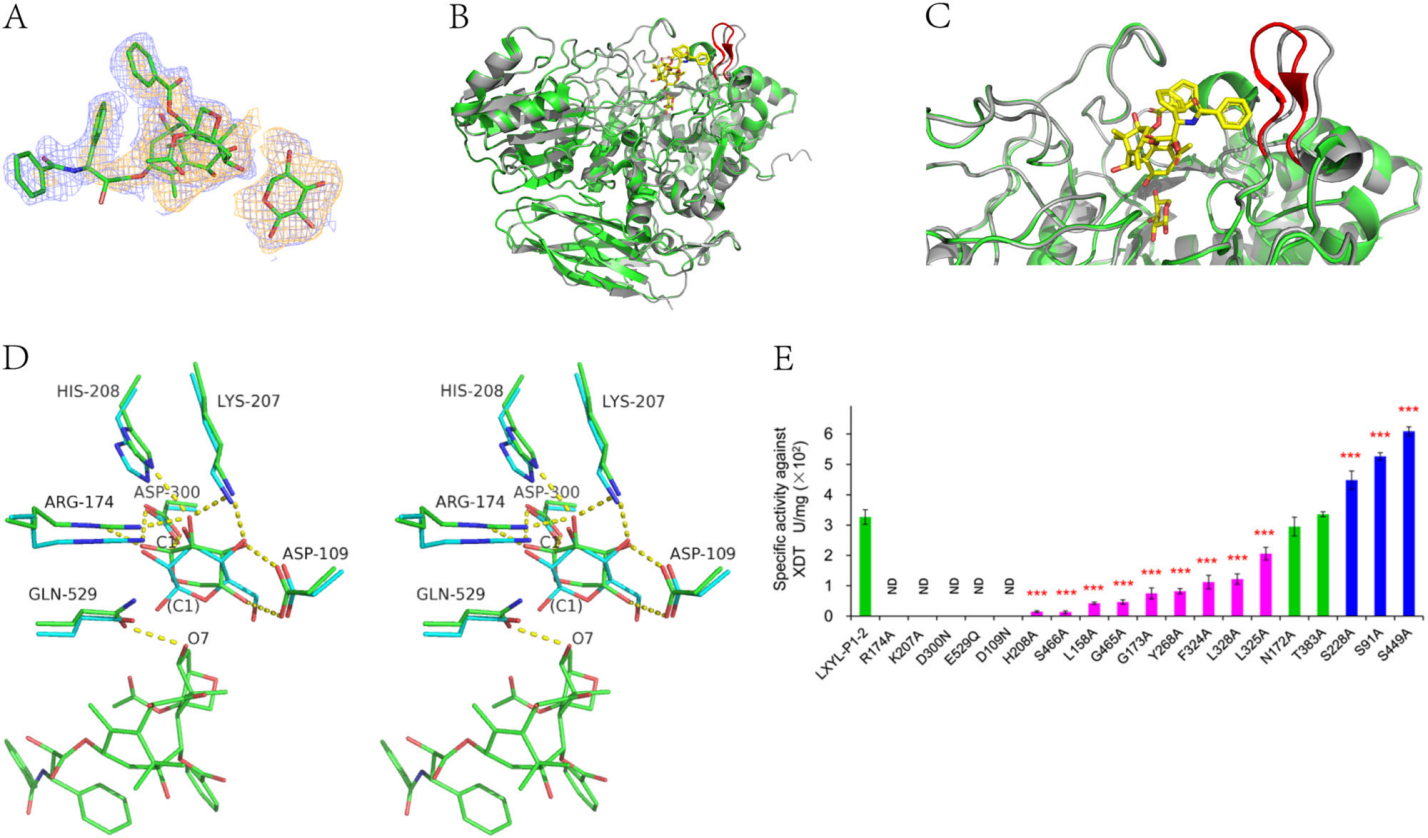
**a** The 2Fo-Fc electron density map (blue, 1.0σ) and Fo-Fc omit map (orange, 3.0σ) for xylose and DT. **b** Comparison of the overall structure of LXYL-P1-2 in substrate free form (gray) and XDT binding form (green). The moving loop Ile^222^-Gln^229^ is shown in red. DT and xylose are shown in a stick model. **c** A big view of comparison of the detailed structure of LXYL-P1-2 in substrate free form (gray) and XDT binding form (green). The moving loop Ile^222^-Gln^229^ is shown in red. DT and xylose are shown in a stick model. **d** Stereo view of the active site comparison of LXYL-P1-2 (green) and *Hj*Cel3A (PDB code 3ZYZ, cyan). The residues from LXYL-P1-2, atom C1 of xylose and atom O7 of DT are labeled. The C1 atom of glucose bound in *Hj*Cel3A is also labeled in brackets. **e** The specific activities of LXYL-P1-2 and its site-directed mutants on XDT. The data represent the means ± s.d., *n* = 3. *P < 0.05 versus LXYL-P1-2 (Control), **P < 0.01 versus LXYL-P1-2 (Control), ***P < 0.001 versus LXYL-P1-2 (Control), One-way analysis of variance was used for data analysis using SPSS 17.0.

To obtain the substrate binding structure, mutant E529Q was co-crystallized with XDT (PDB code 6KJ0). There are two monomers in the asymmetric unit. The electron density clearly shows the existence of xylose and DT (Fig. 2a). The xylose adopts a pyranose configuration. It was surprised to found that the glycosidic bond between xylose and DT was broken in both of the monomers. Although the E529Q mutant did not show catalytic activity in the standard enzymatic essay, it may still have weak activity to digest the glycosidic bond of XDT during the crystallization time of one week. Thus, this structure should be regarded as the enzyme/product complex.

Sequence alignment of LXYL-P1-2 indicates that Asp^300^ and Glu^529^ might act as the catalytic nucleophile and the acid/base residues, respectively. In LXYL-P1-2 structure, the distance of these two residues is 5.5 Å, consistent with the proposed retaining catalytic mechanism in GH3 family. In the substrate free enzyme, Glu^529^ forms hydrogen bond to the side-chain of Arg^218^ and the main-chain NH of Ser^466^. In the complex, the amide group of Gln^529^ rotates about 40 degree to form hydrogen bonds to the side-chain OH of Ser^466^ and the O7 atom of DT. This confirms that Glu^529^ plays as acid/base to attack the glycosidic bond. Other residues remain the same conformations as the free state. The side-chains of Asp^109^, Arg^174^, Lys^207^, His^208^, and Asp^300^ stabilize the xylose by forming hydrogen bonds (Fig. 2d).

**Fig. 3:**
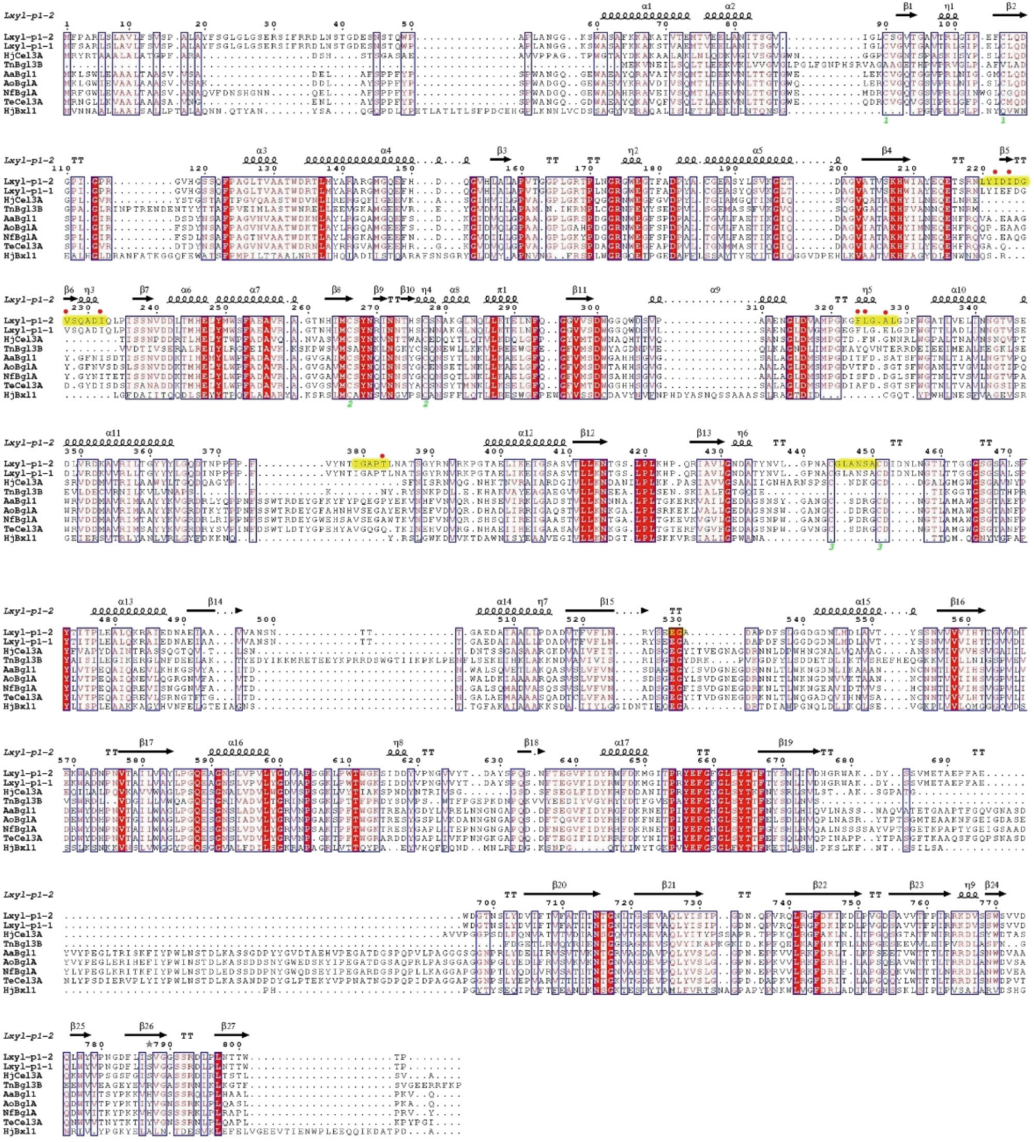
Sequence alignment. Sequence alignment was done on the online sever of T-coffee^23^ and ESPript sever was used for result display^24^. Cysteine in the protein are shown by green figure, and the DT specific residues are shown by red points. XDT binding and catalytic domain are highlight in yellow.

Compared with the free enzyme, the only prominent conformational changes in the overall structure upon XDT binding is the movement of loop Ile^222^-Gln^229^ with the longest distance of 2.5 Å (Fig. 2b, c). This movement suggests that LXYL-P1-2 possesses an open conformation in free enzyme and a closed conformation in substrate binding state. DT molecule binds at a pocket formed by several loops: residues 220–232 and 324–328 from domain 1, residues 379–383 which links domain 1 and 2, and residues 446–450 and 529–530 from domain 2 (Fig. 3). Therefore, domain 1 and domain 2 together make the DT binding site. Besides the hydrogen bond between O7 of DT and Glu(Gln)^529^, no other hydrogen bonds or static electric interactions were observed between LXYL-P1–2 and DT. The hydrophobic environment, contributed by Leu^220^, Ile^222^, Ile^224^, Val^227^, Ile^232^, Phe^324^, Leu^325^, Ala^327^, Leu^328^, Ile^379^, Thr^383^, and Ala^447^ form the hydrophobic wall of DT binding pocket. The modified oligosaccharide chains seem not contact with the substrate, which indicates that the glycosylation does not contribute to the enzymatic activity.

### Site-directed mutations of the XDT binding region

According to the enzyme-substrate complex structure, a number of residues at xylose and DT binding site were subjected to site-directed mutations to inspect their influence to the enzyme activities. Ala-scanning mutations were carried out and the activities of the mutants are summarized in Fig. 2e. Unsurprisingly, the mutations of conserved catalytic residues all showed an inactive or very low active. Deceased activities were observed on F324A, L328A, and L325A mutants, which locate at the recognition area for benzoate group of DT. Mutation S228A destroyed the potential hydrogen bond between OG-Ser^228^ and N-Tyr^221^ (3.0 Å in substrate free structure, 4.5 Å in E529Q), making it easier for loop Ile^222^-Gln^229^ to move. Mutation S449A might provide more hydrophobicity in DT binding pocket. It is surprised that mutation S91A, which hydrogen bonds to Asp^109^, increased the enzyme activity, too. This might indicate that Asp^109^ that interacts with xylose needs more flexibility during catalysis.

## Discussion

Comparison with other GH3 enzymes shows that the crystal structures of LXYL-P1-2 provide the detailed information of xylosidase with the activity to hydrolyze 7-β-xylosyl-10-deacetyltaxol for the first time to our knowledge. Other structures similar to LXYL-P1-2 were found by using Dali server^8^. As expected, the overall fold of LXYL-P1-2 is resembled to some three-domain GH3 members. The RMSD of main-chain atoms is 1.3 Å when superimposing LXYL-P1-2 to the most similar structure *Tn*Bgl3B^9^. In the structure of LXYL-P1-2, eight cis-peptide bonds are found in Asp^109^-Gly^110^, Ala^159^-Pro^160^, Gly^164^-Pro^165^, Lys^207^-His^208^, Trp^209^-Ile^210^, Met^319^-Pro^320^, Ala^381^-Pro^382^, and Leu^419^-Pro^420^. Six of these cis- peptide bonds are conserved in many GH3 members, except for Ala^381^-Pro^382^ that makes a sharp turn involved in DT binding pocket. In addition, two intra-molecular disulfide bonds are formed by Cys^266^-Cys^277^ and Cys^444^-Cys^451^. These disulfide bonds are also conserved in most of GH3 members. Structure comparison also shows that the conformations of the residues around the sugar binding site, including the catalytic nucleophile Asp^300^ and the acid/base Glu^529^ are strictly conserved (Fig. 2d). This suggests LXYL-P1-2 share the common hydrolytic mechanism in GH3 family.

Interestingly, the xylose ring orientation rotated by about 60 degrees compared to the glucoses found in other GH3 enzyme structures as in *Hj*Cel3A^10^. As shown in Fig. 2d, the OH-1,-2,-3 groups of xylose is corresponded to the OH-2,-3,-4 of glucose. The OH-1 of glucose points outwards of the active site, where the +1 group of substrate could be linked. The glucose soaked in crystals mimics the substrate binding state. In LXYL-P1-2, the xylose is generated from the hydrolysis of XDT and represent the product binding state, in which the OH-1 group is away from DT, with the distance between C1 of xylose and O7 of DT being 4.3 Å. If the xylose is orientated as the in *Hj*Cel3, the distance between C1 and O7 would be only 3.0 Å, indicating a good position to form the glycosidic bond. Therefore, the glucose soaked in *Hj*Cel3 crystals might mimic the substrate binding state. In LXYL-P1-2, the xylose is generated from the hydrolysis of XDT and might represent the product binding state. Both in the substrate and the product states, although the C1 atom of xylose in LXYL-p1-2 and the C1 atom of glucose in *Hj*Cel3 are at different positions, both of them are close to the nucleophile residue (Asp300 in LXYL-P1-2 and Asp236 in *Hj*Cel3, with the C1-OD1 distances of 2.7 Å and 2.8 Å).

In the proposed retaining mechanism of glycosidase, a covalent intermediate complex is expected to be formed by the sugar group of substrate and the nucleophile residue. The structures of xylose and glucose described above might depict the two states before and after the covalent intermediate stage. This might be the first report elucidating the rotation of the sugar ring from substrate binding to product forming states.

Besides comparison above, the tetramer formation attracts our attention. The catalytic pocket of each monomer faces to the outside of tetramer. The tetramer formation seems not influence on the active pocket, but contributes to the highly thermal stability of LXYL-P1-2. Two interfaces are defined in the LXYL-P1-2 tetramer. On interface A, His^118^, Tyr^390^, Asn^392^, Arg^394^, Arg^426^, Tyr^436^, Glu^479^, Gln^482^, Asn^489^, and Glu^491^ form a number of hydrogen bonds between the two monomers, while Y^473^-I^486^*, L^478^-Y^436^*, L^456^-V^438^* (*indicates the residue from the neighboring monomer) form hydrophobic pairs. On interface B, Arg^678^-Tyr^363^*, Asp^675^-Lys^66^* form hydrogen bonds. In addition, Van der Waals interactions of Ser^62^-Trp^679^*, Thr^368^-Phe^710^*, and Asn^369^-Val^757^ also contribute to interface too. The interface A seems conserved as in AaBGL (Fig. 1b). However, the modified oligosaccharides are involved in dimer formation^11^. In LXYL-P1-2, there is no glycan modification close to this interface. The interface B was never reported in GH3 family. *Km*BglI^12^ and *Ao*βG^13^ were reported to be a tetramer, but their dimer-dimer interfaces are different from that in LXYL-P1-2.

Compared to other GH3 enzymes, the sequence and structural variations mainly come from the loops, especially those forming the substrate binding pocket (Fig. 3). Loops of residues 220-232, 324–328, 379–383, 446–450, and 529–530 form the DT binding pocket. As shown in the sequence alignment, the first four loops are conserved in LXYL-P1-1 and LXYL-P1-2 but are quite different from other GH3 enzymes. In fact, when superimposing other GH3 enzymes structures with LXYL-P1-2, they may have residues occupy the position of DT binding, such as Val^298^ and Phe^29^ in *Tn*Bgl3B^9^, Trp^37^, Phe^260^, and Trp^443^ in *Hj*Cel3A^10^, Trp^274^, Leu^295^, and Tyr^510^ in *Km*BglI^12^. The DT specificity results from the loop sequences, with the residues contributing hydrophobic side-chains towards the binding DT molecule. This suggests the pocket size, shape and hydrophobic environment are critical to DT recognition. As shown in Fig. 2e, the mutants with large hydrophobic side-chain removed decrease the enzymatic activity. In contrast, mutations of the surrounding residues S91A and S449A, which increase the hydrophobicity, could improve the catalytic ability.

To confirm the importance of the DT-binding loops, we purified the recombinant *Tn*Bgl3B that shares the same sugar binding site but different DT-binding loops, and tested its activity on PNP-Glc, PNP-Xyl, and XDT, respectively. The results showed that although *Tn*Bgl3B exhibited considerable activities on PNP-Glc and PNP-Xyl, the activity on XDT was undetectable (Table 3). In order to find more candidate enzymes with XDT xylosidase activity, we searched the whole genome in EXPASY blast server [https://web.expasy.org/blast/]. No protein except LXYL-P1-1 and LXYL-P1-2, are found with the similar DT-preferred loops. Therefore, LXYL-P1-1 and LXYL-P1-2 seem to be the only available enzymes that could be applied in DT production to date. The activity-increased mutants show the potential of successful engineering of better enzymes in the future.

**Table 3:** Specific activity of *Tn*Bgl3B and LXYL-P1-2 against PNP-Xyl, PNP-Glc and XDT.

	PNP-Xyl (U/mg × 10^4^)	PNP-Glc (U/mg × 10^4^)	XDT (U/mg × 10^2^)
LXYL-P1-2	7.19 ± 0.87	15.59 ± 2.09	3.27 ± 0.24
*Tn*Bgl3B	1.60 ± 0.03***	11.86 ± 0.70***	ND

Values represent mean ± s.d. of triplicates from a representative experiment (*n* = 3 experiment replicates)

ND Not detected

*P < 0.05 vs LXYL-P1−2

**P < 0.01 vs LXYL-P1−2

***P < 0.001 versus LXYL-P1−2 (Control), One-way analysis of variance was used for data analysis using SPSS 17.0

Another key point of the research is the binding site of Taxol in LXYL-P1-2. Due to the poor solubility in water, Taxol is usually formulated in a mixture of cremophor EL and dehydrated ethanol, which however may have severe side effects on patients (Ma and Mumper^14^). Therefore, the specific carrier proteins are needed for developing Taxol delivery system. To date, Nab-paclitaxel (Abraxane^®^, approved by FDA in 2005) has been the first FDA approved taxoid formulation based on albumin nano-delivery systems, and a number of novel Taxol nano-particle formulations are in clinical trials^14^. The structure of LXYL-P1-2 bound with XDT may provide useful information for designing new Taxol binding proteins.

**Fig. 4:**
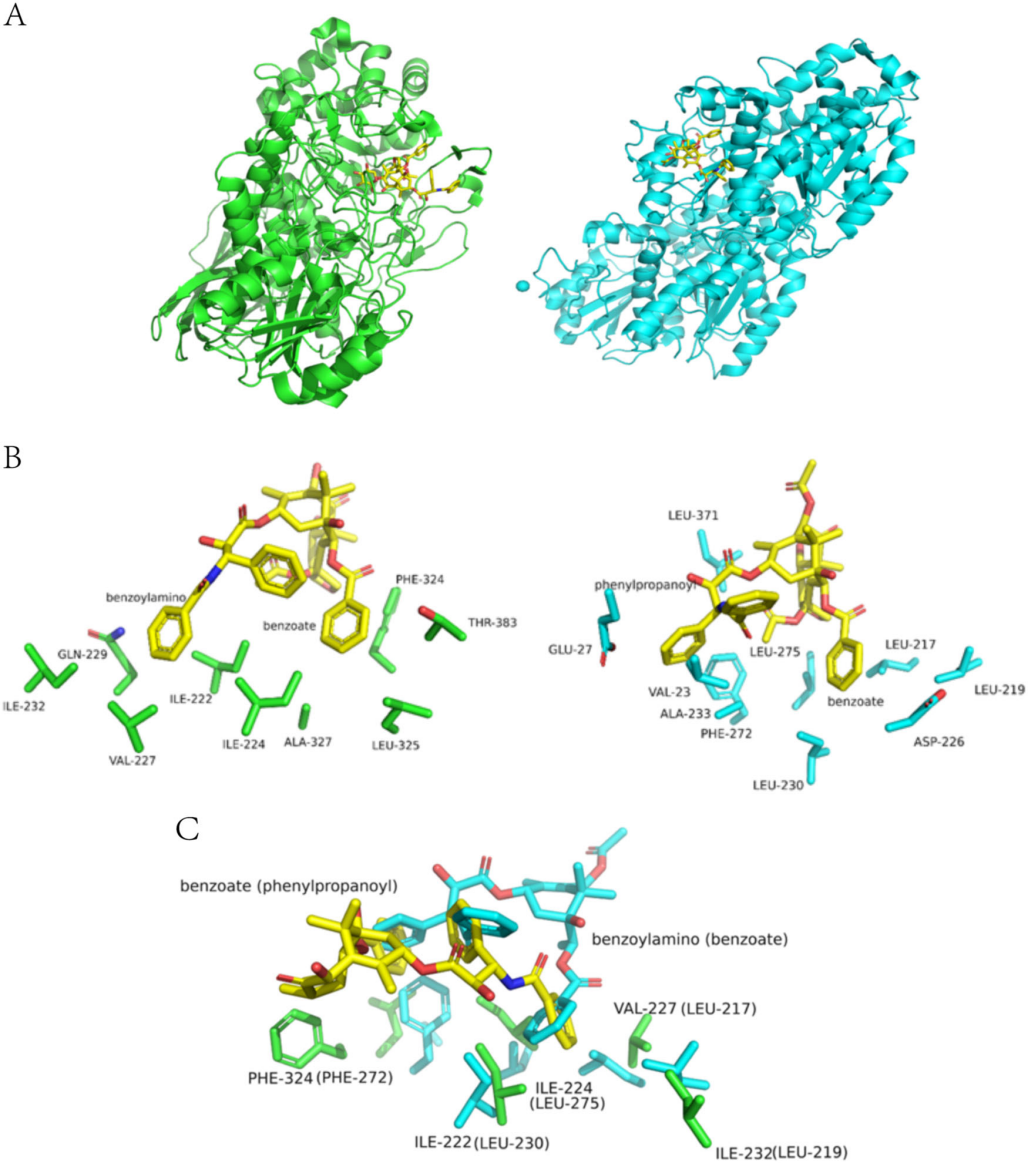
**a** Structure comparison of the overall structure of LXYL-P1-2 (PDB code 6KJ0, green) and tubulin (PDB code 1JFF, cyan). DT and Taxol are shown as a yellow stick model. **b** Structure comparison of DT/Taxol two benzene rings recognition pocket of LXYL-P1-2 (PDB code 6KJ0, green) and tubulin (PDB code 1JFF, cyan). DT and Taxol are shown as a yellow stick model. The residues involved in DT/Taxol binding are shown as stick. **c** Structure superposition of DT/Taxol binding pocket of LXYL-P1-2 (green) and tubulin (cyan). DT is shown as a yellow stick model. Taxol is shown as model color stick. The residues involved in DT/Taxol binding are shown as stick. Label of tubulin are shown in parentheses.

In Protein Data Bank, Taxol is only found in tubulin/Taxol complex with the highest resolution of 3.5 Å. Our study shows the first high resolution structure of Taxol analogs bound in proteins. In order to search the possible common recognition features for DT or Taxol binding, the complexes of LXYL-P1-2/XDT and tubulin/Taxol^15^ are compared. Although the overall structures of LXYL-P1-2 and tubulin are totally different, the conformations of the DT or Taxol main body are superimposable (Fig. 4a, c) and two similar binding pockets could be identified, but with the binding groups are swapped.

As shown in Fig. 4b, loop of residues 220–232 in LXYL-P1-2 forms the first pocket that recognizes the benzoylamino of DT. The non-polar amino acid residues, Ile^222^, Ile^224^, Ile^232^, and Val^227^, form a semi-enclosed hydrophobic pocket, in which the benzoylamino is half surrounded, while the side-chain of Gln^229^ and the main-chain O of Val^227^ form the hydrophilic area at the bottom of the pocket. In the similar way, the pocket for benzoate biding in tubulin is also a semi-enclosed hydrophobic pocket formed by Leu^217^, Leu^219^, Leu^230^, and Leu^275^, while Asp^226^ and His^229^ form the hydrophilic bottom.

Loop324–328 of LXYL-P1-2 forms the second pocket, which is bound with the benzoate of DT. This pocket is formed by the side chain benzene of Phe^324^, the side chain methyl group of Ala^327^, the side chain of Leu^325^, the CG2 atom of Thr^383^. Tubulin has a similar pocket, which is formed by Phe^272^, Ala^233^, Val^23^, and Pro^360^. Instead of binding the benzoate in LXYL-P1-2, however, this binding pocket is for the phenylpropanoyl group binding in tubulin.

Further structural analysis indicates that Ile^222^, Ile^224^, Ile^232^, Val^227^, and Gln^229^ in LXYL-P1-2 have the similar spatial distribution as Leu^217^, Leu^219^, Leu^230^, Leu^275^, and Asp^226^ in tubulin (Fig. 4c). It is interesting that the three benzene rings of DT and Taxol are also in the same spatial position when superposed (Fig. 4c), which partially supports the conserved spatial distribution of binding pockets in Taxol binding. Besides the structural information, the results of our enzyme catalytic experiments demonstrate that Phe^324^ and Leu^325^ in the benzoate recognition region is critical for substrate recognition (Fig. 2d). Therefore, this spatial distribution of hydrophobic residues would make an interface with two pockets specific for Taxol binding. This is the first Taxol analog binding structure except tubulin and might be the only enzyme to catalyze XDT, which may provide useful information for Taxol analogs design.

## Methods

### Materials and strains

The plasmid *pPIC3.5K-lxyl-p1****-****2* was cloned in our lab^5, 16^. The *TnBgl3B* gene (GenBank: ABI29899.1) was synthesized by SynBio Research Platform at Tianjin University (Tianjin, China). Phusion polymerase, restriction enzymes, and T4 ligase were purchased from New England Biolabs (Ipswich, MA). *Escherichia coli* Transeetta (DE3) competent cells and plasmids were purchased from TransGen Biotech (Beijing, China). The pET-28a plasmid was purchased from Novagen (Malaysia). XDT and DT (HPLC purity >99%) were purified in our laboratory. All other chemicals were analytical grade unless otherwise indicated.

### Construction of active-site mutants of LXYL-P1-2 and *TnBgl3B* recombinant strain

The plasmid *pPIC3.5K-lxyl-p1****-****2* was used as DNA template. The L-alanine scanning mutations and other active-site mutations were all obtained by means of the site-directed mutagenesis technique with Phusion High-Fidelity DNA Polymerase (NEB) by using whole-plasmid amplification PCR. All products were sequenced to ensure that no base change other than designed. The plasmids were extracted, linearized with *Sac* I and transformed into the *Pichia pastoris* GS115 competent cells for expression.

The full-length *TnBgl3B* gene was amplified by using forward primer (F: 5′-AAGGATCCATGGAAAAGGTTAACGAGATC-3′) with *Bam*H I site (underlined) and reverse primer (R: 5′-TAGCGGCCGCTTAAGGCTTGAATCTTCTC-3′) with *Not* I site (underlined). The PCR products were digested by *Bam*H I and *Not* I for directional ligation into vector pET-28a. After ligation, the construct was sequenced and transformed into *E. coli* Transeetta (DE3) competent cells for expression.

### Protein expression, purification and activity assay

The heterologous expression and deglycosylation of LXYL-P1-2, as well as the mutants were same to the previous article^5, 16^. *E. coli* cells with pET-28a-*TnBgl3B* recombinant plasmid were grown overnight at 37 °C and 200 r.p.m. in 10 ml Luria-Bertani (LB) medium containing kanamycin (50 μg/ml) in a shaking flask. The overnight culture was suspended in 100 ml fresh LB medium at a final concentration of 1% (v/v), and grown at 37 °C and 200 r.p.m. for 2–3 h until OD_600_ reached 0.8. Then, isopropyl-β-D-thiogalactopyranoside (IPTG) was added at a final concentration of 1 mM and the cell culture was incubated for an additional 20 h at 24 °C and 200 r.p.m. The protein was purified by Ni Sepharose 6 Fast Flow resin and Agilent ZORBAX GF-450 gel-filtration column. The enzyme activities of LXYL-P1-2 were tested as reported previously^5^.

### Crystallization, data collection, and structure determination

LXYL-P1-2 protein was concentrated to 10 mg/ml for crystallization. Both native crystal and complex crystal were grown at 16 °C with the hanging drop vapor diffusion method. For complex crystallization, E529Q protein was mixed with XDT before setting up. Native crystal and complex crystal grew in the solution contain 13% PEG3350, 0.1 M Tris-HCl pH8.5, 0.2% MgCl_2_ and 15% PEG3350, 0.1 M Hepes pH 7.5, 0.2% MgCl_2_, respectively. Diffraction data were collected in BL17U of the Shanghai Synchrotron Radiation Facility (SSRF)^17^. Data were processed using HKL2000^18^. The native crystal diffracted to 2.4 Å (PDB code 6JBS), while the E529Q-XDT complex crystal diffracted to 2.27 Å (PDB code 6KJ0).

The native structure was solved by Phaser^19^ in CCP4 suit^20^ with *Tn*Bgl3B structure (PDB code 2 × 40) as the searching model. The complex structure was solved using native structure as the serching model. Refmac5 was used for strucure refienment^21^. Coot was used for model building^22^. All the strucure figures were prepared using PyMol (https://pymol.org).

### Reporting summary

Further information on research design is available in the Nature Research Reporting Summary linked to this article.

## Supplementary information

Reporting Summary

Peer Review File

## Data Availability

The coordinates of the crystal structures are deposited in Protein Data bank with the entrance codes of 6JBS and 6KJ0.
