# Supplementary material for: Structures of β-glycosidase LXYL-P1-2 reveals the product binding state of GH3 family and a specific pocket for Taxol recognition
Source: Commun Biol. 2020 Jan 10;3:22. doi: 10.1038/s42003-019-0744-4 (PMC6954215; doi:10.1038/s42003-019-0744-4)
Supplement: Supplementary file 2 — Peer Review File [file 42003_2019_744_MOESM2_ESM.pdf]

***Editorial Note:** This manuscript has been previously reviewed at another Nature Research journal. This document only contains reviewer comments and rebuttal letters for versions considered at Communications Biology.*

REVIEWERS' COMMENTS:

Reviewer #1 (Remarks to the Author):

In the revised manuscript the authors solved a new E529Q mutant structure in complex with the substrate/product and also corrected initial wrong space group identification presented in the original manuscript. Therefore, the structural information was considerably improved and enhanced. The authors also satisfactory answered the questions raised by the referees.
The style and grammar of the manuscript are still far from being perfect and failure to predict other GH3 enzymes that could hydrolyze XDT and thus, generalize heir study is somewhat frustrating.
